# Treatment failure and associated factors among people living with HIV on highly active antiretroviral therapy in mainland China: A systematic review and meta-analysis

**DOI:** 10.1371/journal.pone.0284405

**Published:** 2023-05-02

**Authors:** Dandan Niu, Houlin Tang, Fangfang Chen, Decai Zhao, Hehe Zhao, Yushan Hou, Shi Wang, Fan Lyu

**Affiliations:** 1 Division of Epidemiology, National Center for AIDS/STD Control and Prevention, Chinese Center for Disease Control and Prevention, Beijing, China; 2 Division of Treatment and Care, National Center for AIDS/STD Control and Prevention, Chinese Center for Disease Control and Prevention, Beijing, China; Beijing University of Chinese Medicine, CHINA

## Abstract

**Objective:**

Reducing the prevalence of treatment failure among people living with HIV (PLHIV) on highly active antiretroviral therapy (HAART) is crucial for improving individual health and reducing disease burden. This study aimed to assess existing evidence on treatment failure and its associated factors among PLHIV in mainland China.

**Methods:**

We conducted a comprehensive search of PubMed, Web of Science, Cochrane Library, WanFang, China National Knowledge Infrastructure, and SinoMed databases. Relevant studies on treatment failure among PLHIV in mainland China until September 2022 were searched, including cross-sectional, case-control, and cohort studies. The primary outcome was treatment failure, and secondary outcomes were the potential influencing factors of treatment failure. We performed a meta-analysis to pool each outcome of interest, including meta-regression, subgroup, publication bias, and sensitivity analyses.

**Results:**

A total of 81 studies were deemed eligible and included in the final meta-analysis. The pooled treatment failure prevalence among PLHIV in mainland China was 14.40% (95% confidence interval [CI]:12.30–16.63), of which the virological and immunological failure prevalence was 10.53% (95%CI:8.51–12.74) and 18.75% (95%CI:15.44–22.06), respectively. The treatment failure prevalence before and after 2016 was 18.96% (95%CI:13.84–24.67) and 13.19% (95%CI:10.91–15.64). Factors associated with treatment failure included good treatment adherence (odds ratio [OR] = 0.36, 95%CI:0.26–0.51), baseline CD4 counts>200 cells/μL (OR = 0.39, 95%CI:0.21–0.75), HAART regimens containing Tenofovir Disoproxil Fumarate (TDF) (OR = 0.70, 95%CI:0.54–0.92), WHO clinical stage III/IV (OR = 2.02, 95%CI:1.14–3.59) and age≥40 years (OR = 1.56, 95%CI:1.23–1.97).

**Conclusion:**

The prevalence of treatment failure among PLHIV receiving HAART in mainland China was low and tended to decline. Poor adherence, low baseline CD4 count, HAART regimens without TDF, advanced clinical stage, and old age were contributing factors for treatment failure. Relevant intervention programs are needed with increasing treatment adherence through behavioral intervention or precise intervention targeting older adults.

## Introduction

Since the first acquired immune deficiency syndrome (AIDS) case was identified in 1985, China has been faced with the challenge of AIDS prevention and control, as is the same in other countries. According to data from the Chinese Center for Disease Control and Prevention, there were 1.053 million people living with human immunodeficiency virus (PLHIV) and 351,000 cumulative reported deaths in China by the end of 2020 [1]. The number of AIDS-related deaths was 51,250 in 2019, accounting for 71.34% of all infectious disease-related deaths [2]. The disease burden was 139 disability adjusted of life years (DALYs) per million people, which tended to increase according to global burden of disease (GBD) data [3]. Therefore, AIDS prevention and control in China should not be underestimated.

Since the national *Four Frees and One Care* strategy in 2003, the coverage of highly active antiretroviral therapy (HAART) in China has been expanding, from 25% in 2002 to 80% in 2017, and the number of PLHIV on HAART has increased from 13,000 in 2004 to 978,000 in 2020, with 134,000 new PLHIV receiving HAART in 2020 [4]. Although HAART cannot cure AIDS, access to HAART has played a significant role in reducing the incidence and mortality among PLHIV by reconstructing the immune function. Globally, HAART has been widely used as a preventive measure to effectively reduce the number of new infections [5].

China has made some progress in AIDS prevention and control, and the proportion of viral load (VL) suppression among PLHIV receiving HAART has steadily increased and remains at a high level. In 2014, the Joint United Nations Programme on HIV/AIDS (UNAIDS) put forward prevention and control strategies for striving to achieve 90-90-90 by 2020 and 95-95-95 by 2025, meaning that 90% and 95% of PLHIV on HAART needed to achieve virological suppression, respectively. The prevalence of virological suppression (VL<400 copies/ml) in China reached 95.4% in 2021 [6].

Evaluations of the treatment failure and associated factors are conductive to accurate policymaking and improvement of the HAART effect, for example, the timely discovery of adverse reactions to HAART drugs and replacement of appropriate drugs when drug resistance occurs. As the number of PLHIV receiving HAART increases, treatment failure usually occurs due to loss to follow-up, poor adherence, drug resistance, and other factors [7]. A meta-analysis indicated that the average drug resistance prevalence of HIV-1 among PLHIV on HAART was 44.7% (95% confidence interval [CI]:39.3–50.2) in China from 2001–2017 [8], and treatment adherence showed a downward tendency [9]. Moreover, the coronavirus disease 2019 (COVID-19) pandemic in 2020 impeded the maintenance of treatment in a qualitative study owing to travel restrictions, communication obstacles, shortage in personnel, privacy concerns, and insufficient HAART reserves [10]. Social and institutional disruptions have contributed to an increased risk of HAART interruption among PLHIV in China [11]. Thus, it is necessary to clarify the current situation of treatment failure and explore the associated factors.

The World Health Organization (WHO) defines treatment failure among PLHIV as a clinical, immunological, or virological failure [12]. Research has shown that the sensitivity and specificity in assessing the therapeutic effect by clinical and immunological failure were low, while virological failure was more sensitive and effective [13]. In 2013, the WHO recommended VL testing as the gold standard for treatment failure [12].

Treatment failure was likely to cause drug resistance and even death through drug-resistant mutations, and the early detection of treatment failure could reduce the occurrence of drug resistance. Before 2007, there was scarce funding support for VL testing in China; since then, it has been provided through a transfer payment. However, in terms of the limitations of staff and the capabilities of laboratory testing, the proportion of PLHIV receiving VL testing in 2007 was still low at 9.1%, and then increased gradually to 71.3% by 2010 [14]. Because it is not guaranteed that any PLHIV are tested for VL each year, there is a lack of comprehensive evaluation of the HAART effect among PLHIV nationwide.

There have been several studies on treatment failure in other countries. In Ethiopia, a systematic review and meta analysis demonstrated that the treatment failure prevalence was high and affected by treatment adherence, WHO clinical stages, exchange of treatment regimens, and opportunistic co-infections [15]. In resource-limited areas, the CD4+T lymphocyte (CD4) counts and treatment adherence have affected treatment failure [16]. In China, the prevalence of treatment failure in different studies has varied widely, ranging from 0.0% [17] to 42.6% [18], and there were some contradictions in influencing factors among related studies, for example, sex [19, 20]. Our study aimed to understand the current situation of treatment failure in mainland China through a meta-analysis of observational studies to help evaluate treatment effects and formulate targeted intervention strategies.

## Materials and methods

### Literature search

Six databases were used for a comprehensive search to obtain relevant Chinese and English studies on treatment failure among PLHIV in mainland China by September 2022, including PubMed, Web of Science, Cochrane Library, WanFang, China National Knowledge Infrastructure (CNKI), and SinoMed. Database searches were supplemented by citation and manual searches to trace important historical. The search method mainly comprised MeSH and free-word research. The complete search formula was ("People’s Republic of China" OR "Mainland China" OR "China" [MeSH]) AND ("HIV"[MeSH] OR "HIV-1" OR "HIV-2" OR "HIV1" OR "HIV2" OR "HIV infections" [MeSH] OR "Human Immunodeficiency Virus" OR "Acquired Immunodeficiency Syndrome" [MeSH] OR "Acquired Immune Deficiency Syndrome Virus" OR "Acquired Immuno Deficiency Syndrome Virus" OR "Acquired Immuno-Deficiency Syndrome Virus" OR "AIDS") AND ("Antiretroviral Therapy, Highly Active" [MeSH] OR "HAART" OR "Combination Antiretroviral Therapy") AND ("Treatment outcome" [MeSH] OR "Treatment failure" OR "Virological failure" OR "Immunological failure" OR "Clinical failure" OR "Failure" OR "Less CD4 count" OR "Viral load").

### Inclusion and exclusion criteria

All observational studies related to treatment failure among PLHIV receiving HAART in mainland China were included, including cross-sectional, case-control, retrospective, prospective, and ambi-directional cohort studies.

Duplicated studies, studies designed as reviews or meta-analyses, studies with incomplete data or unclear outcome indicators, and studies with a Newcastle-Ottawa Scale Quality Assessment Tool (NOS) quality score less than 6 were excluded.

### Outcome variables

Treatment failure was the main outcome variable. To enhance comparability among different countries, treatment failure was assessed through clinical, virological, or immunological failure, or a combination based on the 2016 WHO guidelines [21]. Specifically, clinical failure referred to new or recurrent clinical event indicating severe immunodeficiency (WHO clinical stage 4 condition) after 6 months of effective treatment, virological failure referred to VL>1000 copies/ml based on two consecutive viral load measurements after 3 months with adherence support, and immunological failure referred to a CD4 counts decrease to or below the treatment baseline value or remained below 100 cells/μl after receiving HAART for 6 months.

The secondary outcomes were the potential influencing factors of treatment failure, including treatment adherence, baseline CD4 counts, HAART regimens, WHO clinical stages, age, and sex. According to the requirements of the *Fourth Edition of the National Free HIV/AIDS Antiretroviral Therapy Manual*, treatment adherence ≥95% indicated good adherence; that is, the “missing medication last week” among PLHIV on HAART was less than once. HAART regimens were mainly first-line antiretroviral drugs, including zidovudine (AZT)/stavudine (D4T)+di-deoxyinosine (DDI)/lamivudine (3TC)+nevirapine (NVP)/efavirenz (EFV) before 2008, AZT/D4T+3TC+NVP/EFV in 2008, tenofovir disoproxil fumarate (TDF)/AZT+3TC+NVP/EFV in 2012 [4].

### Data extraction

All related studies were screened through the title, abstract, and full text, and data information was extracted through full text based on the standardized format of the data extraction file, which was completed by two reviewers independently, and finally, consistent results were obtained. If there were divergent opinions between them, a third reviewer would make the final determination. We contacted the first author of the original study to obtain additional information, if necessary, including any occasions in which important information was missing, or the research method was unclear. Data extraction information mainly included the year of publication, first author, study period, study area, study type, study population, duration of treatment, sample size, number of treatment failures, and the odds ratio (OR) (or risk ratio (RR)/hazard ratio (HR)) value of influencing factors. The study area was divided into eastern, central, and western regions according to the criteria for economic belt classification in *China Statistical Yearbook* issued by the National Bureau of Statistics. The eastern regions include Liaoning, Beijing, Tianjin, Hebei, Shandong, Jiangsu, Shanghai, Zhejiang, Fujian, Guangdong, and Hainan; the central regions include Shanxi, Heilongjiang, Jilin, Anhui, Henan, Jiangxi, Hubei, and Hunan; and the western regions include Inner Mongolia, Guangxi, Shaanxi, Gansu, Qinghai, Ningxia, Xinjiang, Sichuan, Chongqing, Yunnan, Guizhou, and Tibet.

### Quality appraisal

Two reviewers independently evaluated the quality of the included studies and only high-quality studies were included in the meta-analysis. Discrepancies will be discussed and resolved by both reviewers and in consultation with a third reviewer, especially the quality appraisal about non-English articles. The NOS standard was used for the quality assessment of case-control and cohort studies [22], and the adapted version of the NOS was used for the quality assessment of cross-sectional studies [23]. Each tool included three sections. The first section mainly evaluated the selection of the study population, with a total score of 4 points; the second section mainly evaluated the comparability between groups, with a total score of 2 points; and the third section mainly focused on assessing outcomes of interest and statistical methods, with a total score of 3 points. The total NOS score was 9 points, and better quality studies had a higher total score. If one observational study scored below 6 points, it was considered low quality and was excluded from our meta-analysis.

### Statistical analysis

Data information of the included studies, including the year of publication, first author, study type, sample size, and other information, were extracted in Microsoft Excel format, and the meta-analysis, including the pooled treatment failure prevalence and its influencing factors among PLHIV on HAART in mainland China, was completed in R4.1.2. Normality tests were conducted using the Lilliefors method, and data that did not meet normal distribution were converted to arcsine [24]. Heterogeneity of prevalence among different studies was evaluated using Cochrane Q tests and I^2^ statistics. Heterogeneity was significant when I^2^≥50%, and a random effect model was used to pool treatment failure prevalence, OR value, and 95%CI. The sources of heterogeneity could be explored by both meta-regression and subgroup analysis based on the year of publication (<2016 vs. ≥2016), study area (eastern, central, and western), study population (adults vs. children), study type (cross-sectional vs. cohort study), sample sizes (≤1000 vs.>1000), duration of treatment (≥12 months, <12 months, and mixed), and treatment failure criteria (virological, immunological, and mixed) [25]. The bonferroni method was used for pairwise comparisons among multiple groups, and the Mann-Kendall test was used for the time trend test. Funnel plot and Egger’s test were used to evaluate publication bias, and sensitivity analysis was conducted by deleting each study.

Because the OR values usually did not meet the normal distribution, log(OR) values were used to ensure stable data and weaken heteroscedasticity and collinearity. Only the adjusted OR values in the multivariable analysis were pooled, and the RRs or HRs were converted through a formula or directly considered as estimated OR values [26]. Hypothesis testing was 2-sided and an alpha of 0.05 was used to indicate a statistically significant difference in our analysis.

## Results

### Flow diagram

**Fig 1:**
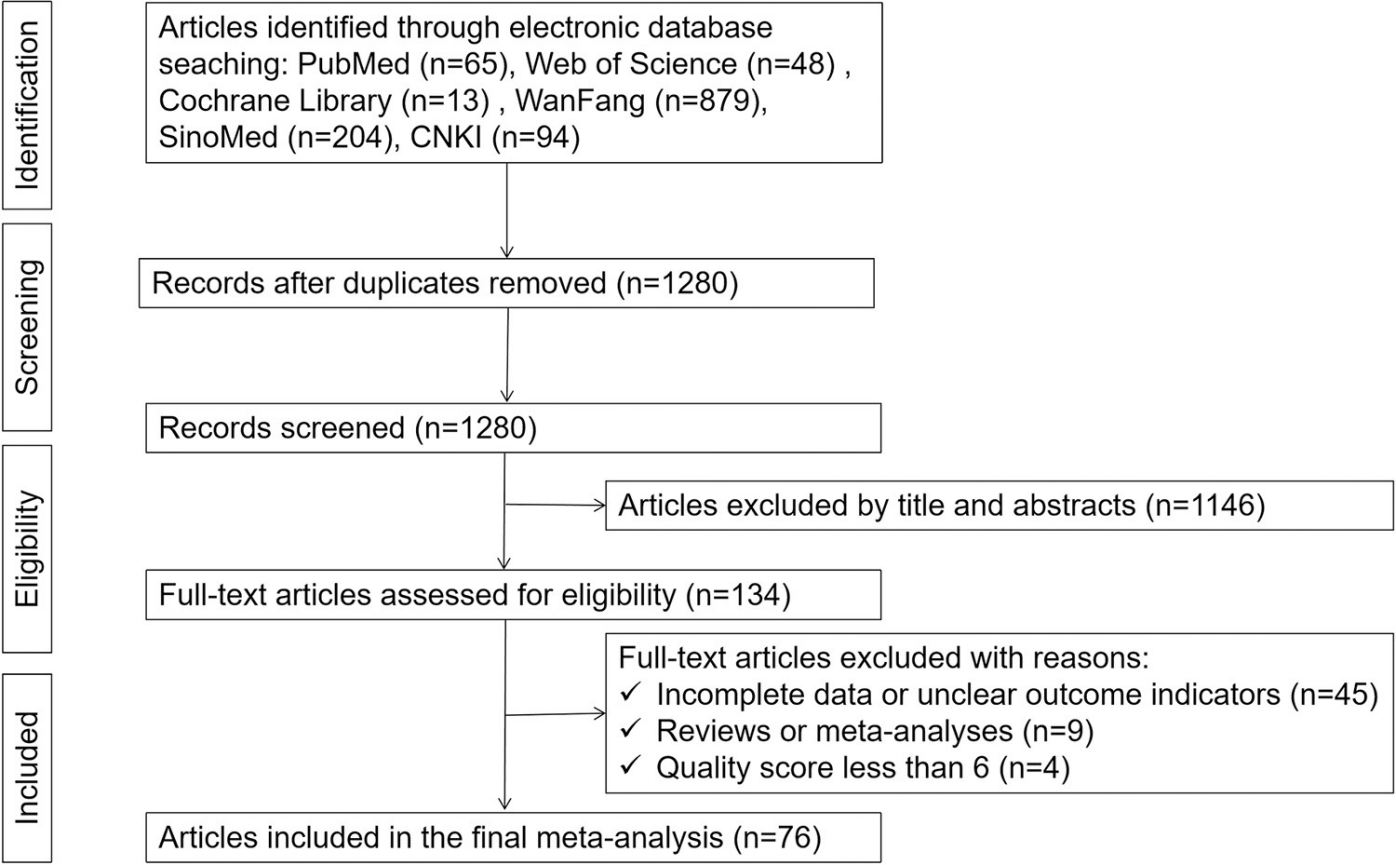
Flow diagram of study retrieval.

We retrieved 1303 articles related to treatment failure among PLHIV on HAART in mainland China by searching the PubMed, Web of Science, Cochrane Library, WanFang, CNKI and SinoMed databases. Among the initial articles, 1280 articles were non-duplicate, of which 1146 were excluded after browsing the title and abstract and confirming irrelevance to treatment failure. A total of 58 articles were excluded through full-text assessment, including nine articles designed as reviews or meta-analyses, 45 articles with incomplete data or unclear outcome indicators, and 4 articles with NOS scores less than 6 points. Finally, 76 articles (81 studies) met the inclusion criteria and were included in the meta-analysis, as shown in the flow diagram of study retrieval (Fig 1).

### Basic characteristics of included studies

A total of 81 studies related to treatment failure among PLHIV on HAART in mainland China from 2007 to September 2022 were included in the meta-analysis, including 29 cross-sectional, 48 retrospective, 2 prospective, and 2 ambi-directional cohort studies, as described in Table 1 (only 20 studies are shown due to limited space). A total of 436,630 PLHIV were included in these studies, with sample sizes ranging from 40 to 223,524. The overall prevalence of treatment failure was from 0.00% to 42.65%, with the virological, immunological, and clinical failure prevalence respectively ranging between 0.00% to 42.65%, 0.52% to 33.31%, 1.49% (only one study) [27]. Studies with NOS scores of 8 or 9 accounted for 79.0%.

**Table 1:** Characteristics of included studies.

First author/Year	Study design	Regions	Study periods	Study population	Treatment duration	Sample sizes	Number of overall treatment failures	Sample sizes	Number of virological failures	Sample sizes	Number of immunological failures	Factors^a^	NOS Scores
Guo M et al/2022 [28]	Retrospective cohort	Hubei	2018–2020	Adults	≥12 Months	2114	197	2114	197	−	−	⑤	8
Wang XD et al/2022 [29]	Retrospective cohort	Hebei	2018–2020	Adults	≥12 Months	642	29	642	29	−	−		9
Zhang Y et al/2022 [30]	Cross-sectional	Sichuan	2016–2019	Mixed	Mixed	223524	29949	223524	29949	−	−		7
Yang RR et al/2021 [31]	Retrospective cohort	Jiangxi	2006–2018	Adults	Mixed	1520	326	−	−	1520	326	①/⑤	9
Ma YX et al/2021 [20]	Retrospective cohort	Liaoning	2011–2019	Adults	Mixed	243	66	−	−	243	66	①/②/③/④/⑤/⑥	9
Luo XL et al/2021 [32]	Retrospective cohort	Sichuan	2016–2018	Mixed	≥12 Months	3038	392	3038	392	−	−	⑥	8
Dai ZZ et al/2021 [19]	Retrospective cohort	Xinjiang	2020	Adults	Mixed	214	38	214	38	−	−	⑤/⑥	7
Wang YQ et al/2021 [33]	Retrospective cohort	Shandong	2012–2017	Adults	≥12 Months	299	47	299	47	−	−	②/⑤	9
Rymj YMT et al/2021 [34]	Prospective cohort	Xinjiang	2016–2020	Adults	≥12 Months	1478	69	1478	69	−	−		9
Guo M et al/2021 [35]	Cross-sectional	Hubei	2019	Adults	Mixed	1665	150	1665	150	−	−	②	8
Lu YQ et al/2021 [36]	Retrospective cohort	Chongqing	2015–2020	Mixed	≥12 Months	3305	348	3305	348	−	−		9
Wang WJ et al/2021 [37]	Cross-sectional	Henan	2020	Mixed	≥12 Months	1389	89	1389	89	−	−		8
Zhang CY et al/2021 [38]	Cross-sectional	Jiangsu	2018–2020	Adults	Mixed	81	18	81	6	81	12		9
Xiao J et al/2020 [39]	Cross-sectional	Guizhou	2016–2017	Adults	Unknown	4343	555	4343	555	−	−		6
Zhao WX et al/2020 [40]	Retrospective cohort	Yunnan	2005–2018	Mixed	≥12 Months	2170	89	2170	89	−	−	③/⑤/⑥	7
He JY et al/2020 [41]	Retrospective cohort	Zhejiang	2006–2019	Adults	Mixed	2971	410	−	−	2971	410		9
Jiang MD/2020 [42]	Retrospective cohort	Shandong	2005–2018	Adults	≥12 Months	486	132	−	−	486	132	②/④	9
Yu HY et al/2020 [43]	Retrospective cohort	Hunan	2018	Adults	≥12 Months	367	28	367	28	−	−		9
Zheng JL et al/2020 [44]	Retrospective cohort	Zhejiang	2015	Mixed	≥12 Months	1605	27	1605	27	−	−		9
Shen X et al/2020 [45]	Cross-sectional	Jiangsu	2019	Adults	Mixed	561	50	561	50	−	−		9

^a^① treatment adherence, ② baseline CD4 counts, ③ ART regimens, ④ WHO clinical stages, ⑤ age, and ⑥ sex.

### Meta-analysis of treatment failure in mainland China

The treatment failure prevalence among 436,630 participants in 81 studies was converted to arcsine data to meet the standard of normal distribution (Lillie test: D = 0.05, P = 0.801). The random effects model was used because of strong heterogeneity (Q = 14814.97, P = 0.000, I^2^ = 99.5%), and the pooled treatment failure prevalence was 14.40% (95%CI:12.30–16.63).

The virological failure proportions of 370657 participants in 63 studies were also converted to arcsine data (Lillie test: D = 0.07, P = 0.613). The random effects model was used because of strong heterogeneity (Q = 9677.08, P = 0.000, I^2^ = 99.4%), and the pooled virological failure prevalence was 10.53% (95%CI:8.51–12.74).

The immunologic failure proportions of 76645 participants in 28 studies were pooled initially due to a normal distribution (Lillie test: D = 0.12, P = 0.434). The random effects model was used because of strong heterogeneity (Q = 6746.82, P = 0.000, I^2^ = 99.6%), and the pooled immunological failure prevalence was 18.75% (95%CI:15.44–22.06). A meta-analysis of clinical failure prevalence was not performed owing to the inclusion of only one study.

### Meta-regression and subgroup analysis

**Fig 2:**
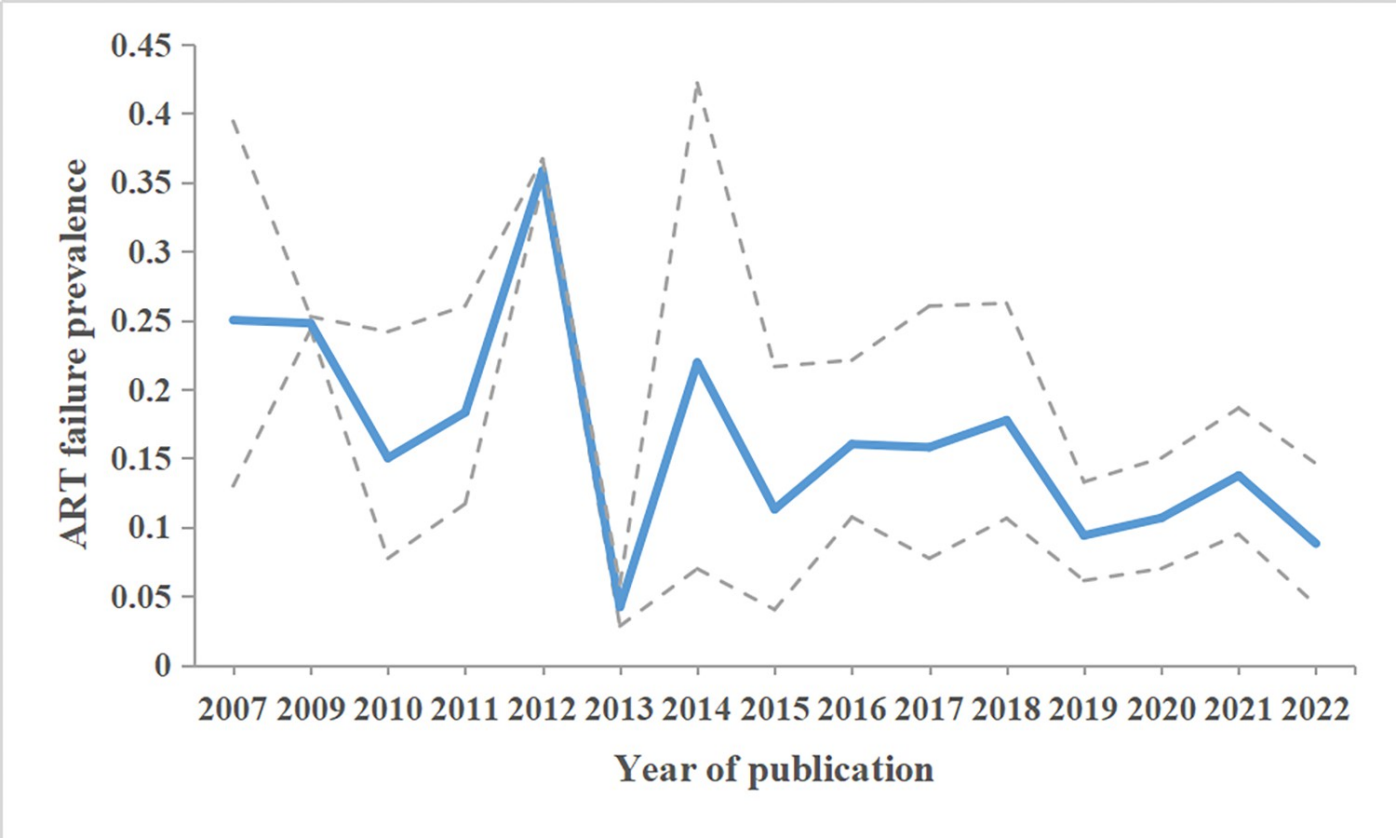
Treatment failure prevalence (95% CI) among PLHIV on HAART in mainland China during the years of publication 2007–2022.

Meta-regression analysis showed that the year of publication (Z = -1.98, P = 0.047) and treatment failure criteria (Z = -2.83, P = 0.005) may lead to heterogeneity in treatment failure prevalence, which could explain 12.79% of heterogeneity based on the coefficient of determination R^2^. The subgroup analysis (Table 2) showed that the pooled treatment failure prevalence before 2016 was higher than that in 2016 and later. In addition, Mann-kendall trend test indicated an overall downward trend during the publication years of 2007–2022 (Z = -2.38, P = 0.018, Fig 2). The pooled treatment failure prevalence was higher among PLHIV with treatment duration <12 months than PLHIV with treatment duration ≥12 months (Bonferroni correction: χ^2^ = 28.43, P<0.0001). The prevalence of immunological failure was higher than that of virological failure (Bonferroni correction: χ^2^ = 1782.70, P<0.0001).

**Table 2:** Subgroup analysis on treatment, virological, and immunological failure prevalence.

Variables	Group	Prevalence of treatment failure (95%CI)	Inter-group heterogeneity	P- values	Prevalence of virological failure (95%CI)	Inter-group heterogeneity	P- values	Prevalence of immunological failure (95%CI)	Inter-group heterogeneity	P- values
Year of publication	<2016	18.96 (13.84–24.67)	3.97	0.046	18.04 (11.91–25.13)	8.76	0.003	17.29 (10.19–24.39)	0.11	0.741
	≥2016	13.19 (10.91–15.64)			8.64 (6.81–10.67)			18.65 (14.83–22.47)		
Duration of HAART	<12 months	18.78 (14.55–23.40)	6.57	0.037	14.92 (8.97–22.08)	3.93	0.140	15.52 (12.83–18.21)	4.61	0.100
	≥12 months	11.20 (7.74–15.20)			8.14 (5.19–11.70)			16.98 (8.75–25.21)		
	Mixed	15.44 (12.63–18.48)			11.09 (8.40–14.10)			20.79 (16.81–24.77)		
Failure standard	CD4 counts below baseline or <100 cells/μl	20.16 (15.77–24.93)	9.22	0.010	−	−	−	−	−	−
	VL>1000 copies/ml	12.43 (10.10–14.95)			−	−	−	−	−	−
	Mixed	14.65 (6.30–25.73)			−	−	−	−	−	−

Meta-regression analysis of the pooled virological failure prevalence showed that the years of publication (Z = -3.39, P = 0.001) and study population (Z = -2.02, P = 0.043) could explain 13.61% of the heterogeneity. The subgroup analysis showed that the pooled virological failure prevalence before 2016 was higher than that in 2016 and later. Meta-regression and subgroup analyses of immunological failure prevalence showed that all factors had no statistical significance, as shown in Table 2.

### Publication bias and sensitivity analysis

**Fig 3:**
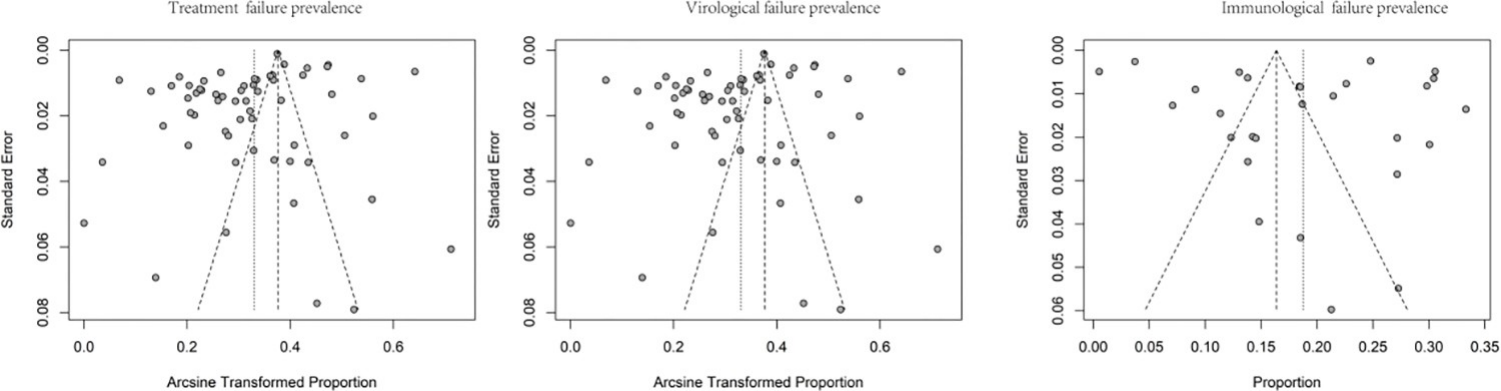
Funnel charts of the pooled treatment, virological, and immunological failure prevalence.

Qualitative analysis of publication bias based on funnel charts (Fig 3) was approximately symmetrical, with several studies beyond the CI. Quantitative evaluation was conducted using Egger’s test and similarly showed that no publication bias existed in the pooled treatment (T = -0.15, P = 0.883), virological (T = -1.75, P = 0.086), and immunological (T = 0.66, P = 0.513) failure prevalence. Sensitivity analysis indicated the stability of the pooled prevalence.

### Associated factors of treatment failure among PLHIV in mainland China

#### Treatment adherence

**Fig 4:**
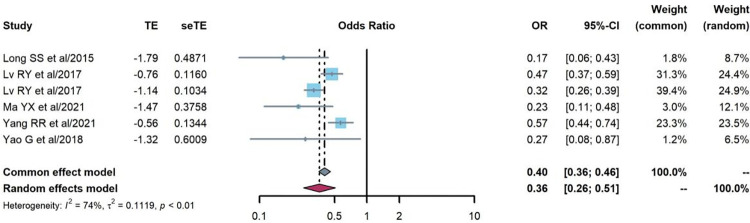
Pooled odds ratios between treatment adherence and treatment failure (good vs. poor adherence).

The association between treatment adherence and treatment failure prevalence was analyzed in six cohort studies [20, 31, 46–48]. The random effects model was used because of strong heterogeneity (Q = 19.29, P = 0.002, I^2^ = 74%), and the odds of treatment failure prevalence among PLHIV with good adherence was lower (OR = 0.36, 95%CI:0.26–0.51), as shown in Fig 4.

#### Baseline CD4 counts

**Fig 5:**
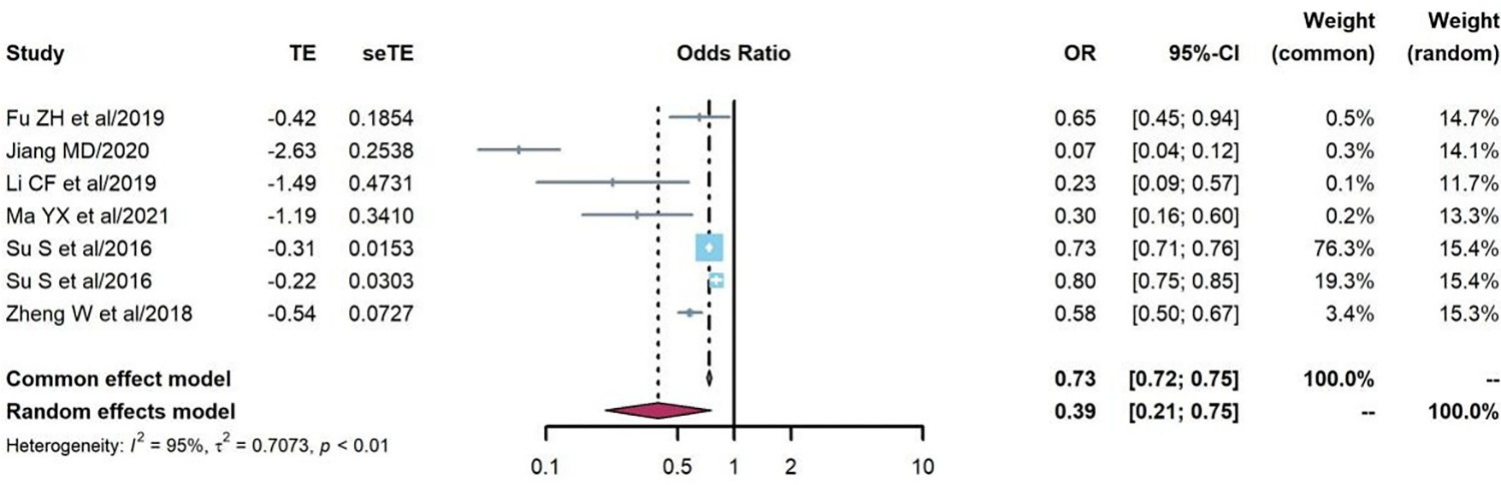
Pooled odds ratios between baseline CD4 counts and treatment failure (CD4 counts>200 cells/μl vs. ≤200 cells/μl).

The association between treatment baseline CD4 counts and treatment failure prevalence was analyzed in seven cohort studies [20, 42, 49–52]. The random effects model was used because of strong heterogeneity (Q = 115.44, P<0.0001, I^2^ = 95%), and the OR of treatment failure prevalence was lower among PLHIV with treatment baseline CD4 counts>200 cells/μl than among those with baseline CD4 counts≤200 cells/μl (OR = 0.39, 95%CI:0.21–0.75), as shown in Fig 5.

#### HAART regimens

**Fig 6:**
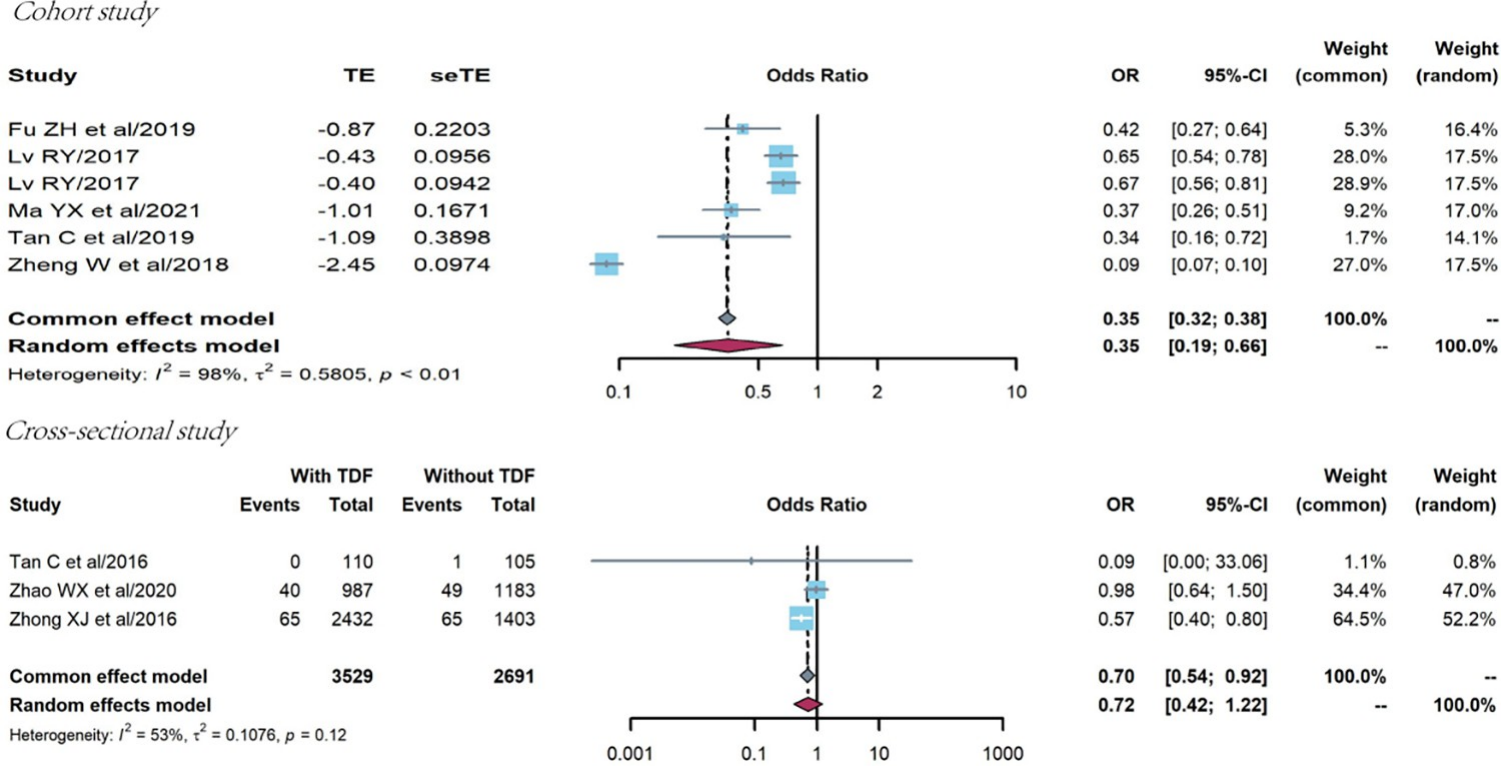
Pooled odds ratio between HAART regimens and treatment failure (with vs. without TDF).

The association between HAART regimens and treatment failure prevalence was analyzed in six cohort studies [20, 47, 49, 52, 53]. The random effects model was used because of strong heterogeneity (Q = 297.99, P<0.0001, I^2^ = 98%), and the OR of treatment failure prevalence among PLHIV receiving HAART regimens with TDF was lower than those without TDF (OR = 0.35, 95%CI:0.19–0.66), as shown in Fig 6.

Three cross-sectional studies were also conducted [40, 53, 54]. The common effect model was used because of low heterogeneity (Q = 4.25, P = 0.119, I^2^ = 53%), and the OR of treatment failure prevalence among PLHIV receiving HAART regimens with TDF was lower (OR = 0.70, 95%CI:0.54–0.92), as shown in Fig 6.

#### WHO clinical stages

**Fig 7:**
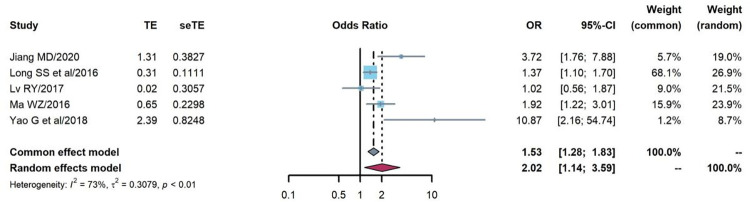
Pooled odds ratio between WHO clinical stages and treatment failure (III/IV vs. I/II).

The association between WHO clinical stages and treatment failure prevalence was analyzed in five cohort studies [42, 46–48, 55]. The random effects model was used because of strong heterogeneity (Q = 14.72, P = 0.005, I^2^ = 73%), and the OR of treatment failure prevalence among PLHIV with WHO clinical stages III/IV was nearly two times higher than those with WHO clinical stages I/II (OR = 2.02, 95%CI:1.14–3.59), as shown in Fig 7.

#### Age

**Fig 8:**
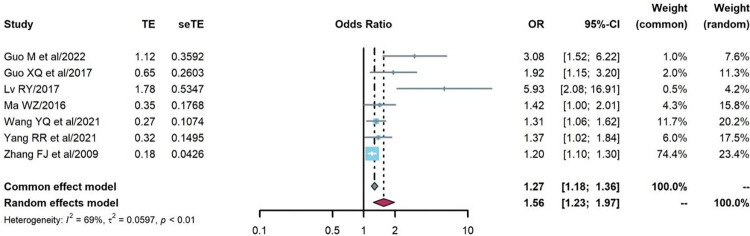
Pooled odds ratios between age and treatment failure (≥40 vs. 15–40 years).

The association between age and treatment failure prevalence was analyzed in seven cohort studies [28, 31, 33, 47, 55–57]. The random effects model was used because of strong heterogeneity (Q = 19.42, P = 0.004, I^2^ = 69%), and the OR of treatment failure prevalence among PLHIV aged over 40 years was higher than those aged 15–40 years (OR = 1.56, 95%CI:1.23–1.97), as shown in Fig 8.

#### Sex

**Fig 9:**
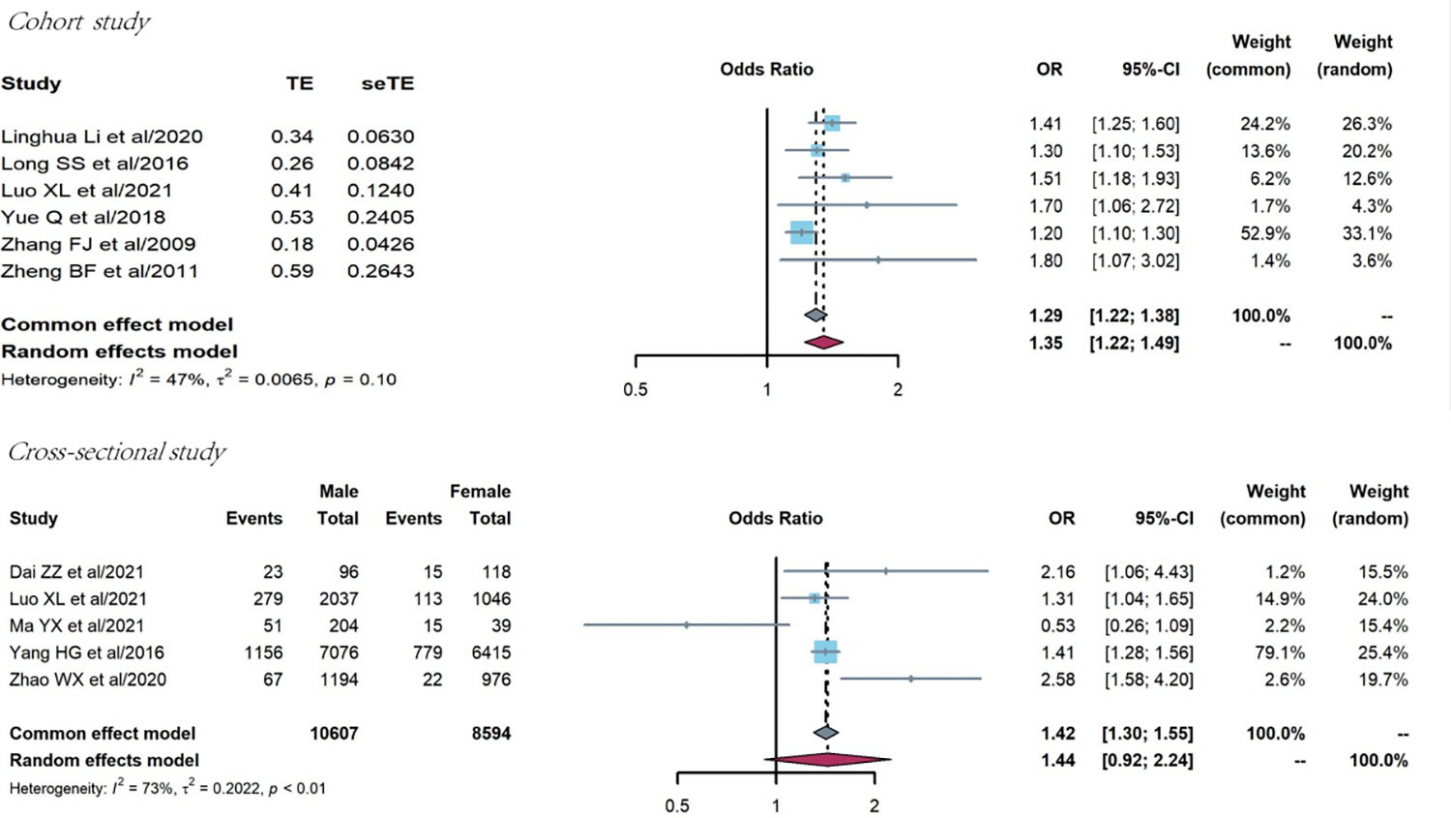
Pooled odds ratios between sex and treatment failure (men vs. women).

The association between sex and treatment failure prevalence was analyzed in six cohort studies [32, 46, 57–60]. The common effect model was used because of low heterogeneity (Q = 9.37, P = 0.095, I^2^ = 47%), and the OR of treatment failure prevalence among men was higher (OR = 1.29, 95%CI:1.22–1.38), as shown in Fig 9.

Five cross-sectional studies were conducted [19, 20, 32, 40, 61]. The random effects model was used because of strong heterogeneity (Q = 14.62, P = 0.006, I^2^ = 73%), and the OR of treatment failure prevalence between men and women had no significant statistical difference (OR = 1.44, 95%CI:0.92–2.24), as shown in Fig 9.

## Discussion

Exploring the current situation of treatment effects and its influencing factors is of great practical significance for further reducing the prevalence of treatment failure. It is well known that treatment failure is likely to increase the risk of drug-resistant strain evolution and HIV-related deaths. Our meta-analysis comprehensively evaluated the treatment failure prevalence among PLHIV receiving HAART in mainland China for the first time. The overall prevalence of treatment failure was low and showed a downward trend. In addition, treatment adherence, baseline CD4 counts, treatment regimens within TDF, WHO clinical stages, and age were associated with treatment failure. Our findings provide scientific evidence for further improvement of HAART strategies in China.

The overall treatment failure prevalence among PLHIV in mainland China was at a low level. Along with the rapid increase in HAART coverage, a continuously low level of VL among PLHIV meant that the risk of HIV transmission was reduced, which has public health significance for AIDS prevention and control in China. Individually, maintaining the treatment failure prevalence at a low level helps prolong survival time. The pooled treatment failure prevalence in mainland China (14.4%) was lower than that in Haiti (15.0%) [62], Ethiopia (15.3%) [15], and Uganda (43.0%) [63], the regional differences among which may be related to treatment failure criteria, sample sizes, social and economic conditions, and testing factors (such as the availability of medical facilities and skills of health workers, etc.). Specifically, the failure prevalence in Ethiopia was pooled based on a smaller sample size (22,849), while the socio-economic conditions in Ethiopia were poorer, which may lead to differences from our results.

The year of publication and the criteria for treatment failure may be important sources of heterogeneity in the prevalence of treatment failure among different studies. The treatment failure prevalence showed a downward trend over the year of publication, which was in accordance with the steady increase in the proportion of virological suppression among PLHIV receiving HAART in China [64]. This achievement may be related to the effective implementation of the Test and Treat strategy in 2016 and the continuous optimization and personalization of free HAART drug regimens. Regarding treatment failure criteria, CD4 counts have become an important basis for HAART initiation and evaluation of treatment effects with the popularization of detection technology since 2005. Free VL testing in China began in 2008, and its coverage prevalence has remained at >90% since 2012 [64], which has become the gold standard for evaluating the effectiveness of HAART [4]. Subgroup analysis showed that the pooled immunological failure prevalence was higher than the virological failure prevalence, which may be related to the lower positive predictive value and greater misclassification based on CD4 counts. More than 80% of the heterogeneity may be caused by testing factors (such as testing process or reagents, sampling time or location, or operating skills) and individual factors (such as the severity of illness or treatment adherence). Because variables in different groups in each study was difficult to unify and analyze, meta-regression analysis allowed the existence of residual heterogeneity.

Treatment adherence, baseline CD4 counts, treatment regimens, WHO clinical stages, and age were factors associated with the treatment failure among PLHIV. Treatment failure largely depended on poor treatment adherence, which could be explained by the fact that poor adherence contributed to the development of resistant varieties and finally caused the failure of current medications. In addition, PLHIV with poor adherence were exposed to CD4 count reduction and increased VL [65]. Treatment adherence is related to variables such as demographic characteristics, treatment factors, and disease characteristics [66]; thus, taking measures to increase treatment adherence, such as simplifying the regimens, health education, directly observed therapy (DOT), and motivational interviewing, may contribute to reducing treatment failure [67]. The treatment failure prevalence was higher among PLHIV with lower baseline CD4 counts, which can be explained by slower immune function recovery [68]. PLHIV with severe immune impairment were prone to maintain high VL for a long time and suffer from opportunistic infections [69], and lower baseline CD4 counts may be associated with a reduced CD4 cell response to HAART [70]. Hence, early treatment is an important measure to reduce treatment failure. The treatment failure prevalence was higher among PLHIV with advanced WHO stages, which is consistent with other studies [15] and can be explained by the higher prevalence of loss to HAART among individuals with severe diseases [71].

The prevalence of treatment failure among PLHIV receiving HAART regimens containing TDF was lower. TDF, the first-line drug recommended by the WHO guidelines for AIDS treatment, was also listed as the first-line drug in the *Third Edition of the National Free HIV/AIDS Antiretroviral Therapy Manual* issued in 2012 in China. The proportion of TDF used in China increased from 1.1% in 2010 to 60.6% in 2014 [72], and our findings similarly showed the effectiveness of TDF. The prevalence of treatment failure was higher among PLHIV patients over 40 years old, which may be related to thymus volume atrophy [73], lower CD4 counts, and worse immune recovery [74]. Therefore, the recovery of immune function among older PLHIV requires special attention. The relationship between treatment failure and sex was different in cohort and cross-sectional studies, and more studies concluded that the treatment failure prevalence among men was higher, which may be attributed to worse treatment adherence [75], lower CD4 levels [76], and worse drug biological responses [77]. However, further studies, especially randomized controlled trials, are needed to verify their exact relationships.

This study had some limitations. First, the pooled prevalence of treatment failure mainly included studies about virological and immunological failure, which might biased the overall prevalence of treatment failure. Second, although meta-analysis using the pooled adjusted OR could obtain more reliable results, the differences in the adjustment strategy of confounding factors in the original studies would increase the heterogeneity among independent studies and eventually affect the authenticity of the pooled effect values. Therefore, on the premise that there would be enough homogenous studies, the prevalence in original studies with the same adjustment strategy for confounding factors should be pooled in the future to provide more accurate evidence for causal inference. Third, gene resistance, opportunistic infections, and changes in treatment regimens were not considered as influencing factors in our analysis because of fewer studies, which may also affect treatment failure. Regarding the pooling of prevalence, large heterogeneity is a common phenomenon at present, and more sophisticated methods need to be developed in the absence of a unified solution to heterogeneity.

In conclusion, the treatment failure prevalence among PLHIV receiving HAART in mainland China was generally low and showed a downward trend. Our findings also indicated that treatment effects could be further improved by strengthening treatment adherence, early treatment, optimizing HAART regimens, and precise intervention of treatment targeted for older adults.

## Supporting information

S1 Checklist PRISMA 2020 checklist. (DOCX)
